# Automated Fluorescence Lifetime Imaging High-Content Analysis of Förster Resonance Energy Transfer between Endogenously Labeled Kinetochore Proteins in Live Budding Yeast Cells

**DOI:** 10.1177/2472630318819240

**Published:** 2019-01-10

**Authors:** Wenjun Guo, Sunil Kumar, Frederik Görlitz, Edwin Garcia, Yuriy Alexandrov, Ian Munro, Douglas J. Kelly, Sean Warren, Peter Thorpe, Christopher Dunsby, Paul French

**Affiliations:** 1 Photonics Group, Department of Physics, Imperial College London, London, UK; 2 Francis Crick Institute, London, UK; 3 RIKEN Center for Biodynamic Systems Research, Kobe, Japan; 4 Garvan Institute of Medical Research, University of New South Wales, Sydney, New South Wales, Australia; 5 Queen Mary University of London, London, UK; 6 Centre for Pathology, Imperial College London, London, UK

**Keywords:** fluorescence lifetime imaging, high-content analysis, budding yeast, kinetochore protein interactions, FRET

## Abstract

We describe an open-source automated multiwell plate fluorescence lifetime imaging (FLIM) methodology to read out Förster resonance energy transfer (FRET) between fluorescent proteins (FPs) labeling endogenous kinetochore proteins (KPs) in live budding yeast cells. The low copy number of many KPs and their small spatial extent present significant challenges for the quantification of donor fluorescence lifetime in the presence of significant cellular autofluorescence and photobleaching. Automated FLIM data acquisition was controlled by µManager and incorporated wide-field time-gated imaging with optical sectioning to reduce background fluorescence. For data analysis, we used custom MATLAB-based software tools to perform kinetochore foci segmentation and local cellular background subtraction and fitted the fluorescence lifetime data using the open-source FLIMfit software. We validated the methodology using endogenous KPs labeled with mTurquoise2 FP and/or yellow FP and measured the donor fluorescence lifetimes for foci comprising 32 kinetochores with KP copy numbers as low as ~2 per kinetochore under an average labeling efficiency of 50%. We observed changes of median donor lifetime ≥250 ps for KPs known to form dimers. Thus, this FLIM high-content analysis platform enables the screening of relatively low-copy-number endogenous protein–protein interactions at spatially confined macromolecular complexes.

## Introduction

Automated high-content analysis (HCA) is widely used in drug discovery^1^ and increasingly in basic life sciences research, for example, for phenotypic screens. HCA has the potential to improve the statistical robustness of smaller-scale biological studies that are typically undertaken using manual cell microscopy, with the ability to automatically image and analyze hundreds to thousands of fields of view (FOVs), enabling significant averaging over experimental noise (including heterogeneity in the biological signal) and reduction of operator bias. Furthermore, analysis of multiwell plate data (with sample replicates and experimental controls) can often highlight systematic errors and thus improve the experimental design. We and others have realized the benefits of HCA for studies based on fluorescence lifetime imaging (FLIM), particularly focusing on its application to read out Förster resonance energy transfer (FRET) of genetically expressed biosensors and fluorescence protein-labeled protein interactions. FLIM provides an inherently ratiometric spectroscopic readout that can contrast different molecular species or read out changes in the local fluorophore environment (including FRET) without requiring spectrally resolved measurements or calibration. Lifetime-based readouts are relatively insensitive to fluorophore concentration, to excitation/detection efficiencies, and to scattering and absorption in the sample or instrument,^2^ and so are useful for assays in cells and biological tissue. While FLIM can provide intrinsic readouts utilizing endogenous fluorophores, for example, enabling the mapping of cellular metabolic changes^3,4^ or stem cell differentiation,^5,6^ and has been implemented with HCA,^7^ it is most commonly applied to exogenous fluorophores. These include small-molecule dyes that stain specific cellular compartments or are conjugated to antibodies, typically used with fixed samples or fluorescent proteins (FPs) encoded by plasmids, which are often employed for live cell imaging.

FLIM can be used to map the physical or chemical environment of exogenous fluorophore probes. FPs may be used, for example, to sense the local lipid phase of cell membranes^8^ or calcium concentration,^9^ and FLIM may be applied to map variations in FRET efficiency, where the close proximity (<~10 nm) of an acceptor fluorophore results in a reduction of the donor fluorescence lifetime. FLIM FRET can be applied with dyes using antibody labeling, for example, to patient-derived tissue arrays to report receptor dimerization,^10^ but is most commonly applied to genetically expressed FP-based FRET biosensors. The latter include “single-molecule FRET biosensors” that incorporate both donor and acceptor fluorophores and therefore present changes in FRET efficiency and donor lifetime upon binding (or being cleaved by) their analyte (e.g., Aoki et al.^11^). For example, FP FRET biosensors can report changes in concentrations of cell signaling molecules such as IP3,^12^ PIP2,^13^ calpain,^14^ and caspase 1,^15^ or ions such as calcium,^16,17^ potassium,^18^ and chloride.^19^ FLIM FRET has also been applied to map interactions of FP-labeled proteins, such as binding, oligomerization, or posttranslational modification—either as endpoints in fixed cells or as dynamic events in time-course measurements of live cells.^20–22^ Gadella^23^ gives an overview of FLIM FRET and its application to study protein interactions.

Automating FLIM FRET can provide the advantages of HCA outlined above, enabling high-throughput assays, for example, of cell signaling processes, and several FLIM HCA platforms have been developed to date. To achieve reasonable throughput, it is necessary to minimize the total acquisition time for each image, including focusing and moving between FOVs. To achieve this with excitation powers compatible with live cell imaging, wide-field FLIM detection can be employed, with optical sectioning realizable using a spinning Nipkow disc.^8,24^ The first reported “unsupervised” FLIM HCA of multiwell plate sample arrays utilized frequency-domain lifetime readouts of FRET in a wide-field (nonsectioning) microscope.^25^ This was followed by our automated multiwell plate optical sectioning FLIM HCA instrument (utilizing wide-field time-gated imaging with a Nipkow disc scanner) that acquired FLIM data with a typical mean acquisition time of ~10 s per FOV. Subsequently, a wide-field (nonsectioning) frequency-domain FLIM HCA system was applied to screen for posttranslational modifications (tyrosine phosphorylation) using FRET.^26^ Automated FLIM and FRET has also been implemented using laser scanning microscopes with time-correlated single-photon counting (TCSPC).^27^ In general, the sequential pixel acquisition makes this approach slower than wide-field FLIM approaches. However, it has been applied successfully in histology, for example, to quantify HER2/HER3 dimerization.^28^

To widen access to wide-field time-gated FLIM HCA, we have subsequently developed an open platform,^29^ openFLIM-HCA, which utilizes wide-field time-gated imaging with open-source software tools for data acquisition^30^ and analysis.^31^ We have applied this approach to FRET assays of intracellular FRET biosensors of calcium dynamics^32^ and small GTPase activation,^33^ and to assays of protein interactions including HIV-1 Gag oligomerization,^34^ SUMOylation of FOXM1,^32^ and a screen for the binding partners of MST-1 among the RASSF protein family.^35^

As this platform matures, we have explored the extent to which it can provide quantitative readouts. The precision to which fluorescence lifetimes can be measured is a function of the number of photons detected,^36^ and wide-field time-gated FLIM provides a means to rapidly acquire signal from multiple pixels in parallel.^37^ The minimum change in fluorophore lifetime required for a reliable assay, for example, of FRET, depends on the numbers of photons detected and also on the biological heterogeneity and the reproducibility of the biological sample preparation. Where transient transfection of FP constructs is employed for intermolecular FRET, the variation in FP expression can be a source of uncertainty in the measurement. The use of stable cell lines is therefore recommended. With reasonably bright FP-based FRET biosensors, as is typical of overexpressed FP-based constructs, the baseline variation of donor fluorescence lifetime can be <20 ps.^30^ While this is encouraging, there remain significant considerations for FLIM FRET assays using FPs. One issue is the artifacts arising from the low rotational mobility of FP, which means that the orientation factor between the donor and acceptor fluorescence dipoles is not dynamically averaged over the fluorescence decay, which is the underlying assumption of standard FRET analysis (i.e., where <κ^2^> = 2/3). This has recently been explored in detail,^38^ and we have developed an analysis to quantify and correct FP-based FLIM data based on a model of fluorophores that are effectively static during their fluorescence decay.^39^ A second issue of concern is that assays based on overexpressed FP constructs can be subject to biological artifacts as the FP constructs compromise the normal functioning of the cell. This consideration suggests that assays of protein interactions should preferably be undertaken with labeled endogenous proteins, which can be realized using tools such as the CRISPR/Cas nuclease.^40^ However, while assays of labeled endogenous proteins may provide more biologically realistic readouts, the signal-to-noise ratio (S/N) can be significantly reduced for low-copy-number proteins compared with assays utilizing overexpressed FP constructs.

**Figure 1.**
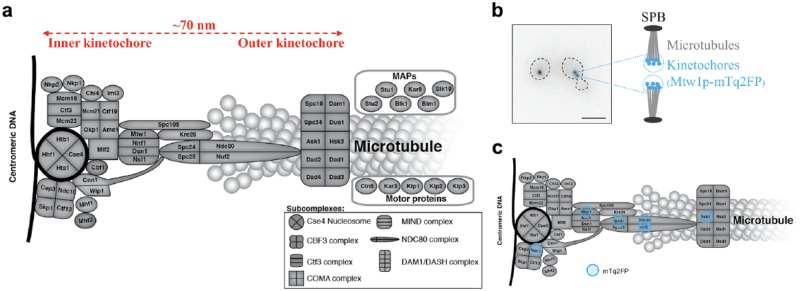
Budding yeast kinetochore. (**a**) The kinetochore is a multiprotein complex comprising various types of KPs that belong to several subcomplexes, together with some microtubule-associated proteins (MAPs) and motor proteins. The architecture of the kinetochore can be divided into three domains: the inner kinetochore is in direct contact with the centromeric DNA; the outer kinetochore interacts with the microtubule; the central kinetochore serves as a bridge between the other two domains. During the metaphase of mitosis, the KPs at each kinetochore span a length of ~70 nm along the kinetochore–microtubule axis. (**b**) Under conventional fluorescence microscopy, the multiple kinetochores (16, 32, or 64, depending on the cell cycle stage and the ploidy) cluster into one or two diffraction-limited foci. For example, in the metaphase/anaphase cell shown on the right-hand side of the micrograph, the fluorescently tagged kinetochores containing Mtw1p-mTq2FP appeared as two foci, each of which contained multiple kinetochores linked to microtubules nucleated from a single spindle-pole body (SPB). Scale bar: 5 µm. (**c**) We chose six KPs (Ndc10p, Mtw1p, Spc24p, Ndc80p, Nuf2p, Ask1p) to be tagged by the FRET donor mTq2FP, respectively, for the FLIM experiments in this study; see proteins with blue circles, which are representative of KPs along the kinetochore–microtubule axis.

Here we explore the limits of our FLIM HCA platform, exploring an assay to screen for protein interactions at budding yeast kinetochores. The budding yeast kinetochore is a highly structured multiprotein complex assembled on each centromere of the chromosomes, which connects them to the spindle microtubules. Thus, kinetochores play vital roles in ensuring faithful chromosome segregation during cell division. The budding yeast kinetochore proteins (KPs) are structurally and functionally well conserved with those in the human kinetochore.^41–43^ A budding yeast kinetochore is comprised of >60 types of proteins, which are organized into various subcomplexes that span a length of ~70 nm along the kinetochore–microtubule axis during the metaphase of mitosis (**Fig. 1a**). The copy number of KPs has been estimated from the fluorescence intensity of endogenously tagged KPs;^44–48^ these data suggest that there are 1 to ~20 KPs per kinetochore depending on the protein in question.^49^ In general, outer KPs (those adjacent to the microtubules) have higher copy numbers than their inner kinetochore counterparts (adjacent to the centromeric DNA).^44^ Depending on the cell cycle stage and ploidy, a budding yeast cell contains 16, 32, or 64 kinetochores, which cluster into one or two diffraction-limited foci when imaged with wide-field fluorescence microscopy (**Fig. 1b**). This clustering makes it challenging to study kinetochore architecture and protein interactions within the kinetochores, and such studies have been mainly performed with genetic and biochemical methods as well as electron microscopy and tomography (see the review by Biggins and references therein^42^). However, optical microscopy methods have made significant contributions,^44,48,50^ including intensity-based FRET microscopy probing the nanoscale organization of endogenously labeled KPs.^51–53^ To the best of our knowledge, we report here the first application of FLIM FRET to study budding yeast kinetochores and the first application of automated FLIM HCA to budding yeast. We note that FLIM FRET utilizing TCSPC with manual data acquisition has been applied to study other FP-labeled endogenous proteins in budding yeast^54–56^ and also to study human kinetochores in vitro^57^—albeit imaging U2OS cells expressing a FRET biosensor fused to the outer KP Hec1, which presents a relatively high abundance per fluorescent focus at its endogenous level.^58^ This approach was recently extended to TCSPC FLIM FRET of the interaction of Ndc80 and kinetochore microtubules in human U2OS cells stably expressing Nuf2 (a subunit of the NDC80 complex) labeled with mTq2FP, which underwent FRET with fluorescein arsenical hairpin binder (FlAsH) bound to a tetracysteine motif labeling endogenous β-tubulin.^59^

We designed the methodology presented here to address the low S/N resulting from the low copy number of endogenous KPs and the strong background of cellular and culture media autofluorescence. Automated imaging with our multiwell plate FLIM microscope controlled by µManager^60^ enabled the unsupervised acquisition of multiple FOVs for each yeast strain, permitting the averaging over experimental noise derived from both instrumentation and biological variability. To validate this approach, we constructed budding yeast strains where KPs were endogenously labeled with donor and acceptor FPs: cyan fluorescent protein mTurquoise2 (mTq2FP)^61^ and yellow fluorescent protein (YFP),^62^ respectively, along with corresponding donor-only negative controls. FLIM HCA measurements indicated the presence of FRET between the donor and the acceptor only when they were tagged to KPs that are previously known to form dimers, suggesting that our methodology provides a reliable way to read out protein associations and likely interactions within the budding yeast kinetochores despite the low S/N. Ultimately, we aim to apply our FLIM HCA methodology to studying interactions between cell cycle regulators and KPs, so as to better understand the signaling events at the kinetochores that when misregulated can to lead to aneuploidy, which is related to many severe diseases in humans, including cancer.^63,64^

## Materials and Methods

### Yeast Strains and Plasmids

The budding yeast *Saccharomyces cerevisiae* strains and plasmids used in this study are listed in **Supplemental Tables S1 and S2**, respectively.

### Yeast Strain and Plasmid Construction

We constructed the yeast strains using standard techniques, and standard yeast growth medium including 2% (w/v) of the indicated carbon source.^65^ To construct haploid strains with one FP gene (mTq2FP or YFP) labeling one KP gene, we used homologous recombination to insert DNA encoding a four-amino-acid (poly-alanine) linker and a fluorophore immediately 3′ to the KP gene in the genomic locus (**Suppl. Fig. S1a**). The construct included 50-base-pair (bp) homology regions at the 5′ and 3′ ends and a selectable marker (encoding hygromycin resistance or histidine prototrophy) to enable selection. We transformed this construct into yeast using a standard high-efficiency lithium acetate (LiOAc) method.^66^ This results in KP with a C-terminus FP label. We confirmed the DNA sequence of all constructs by amplification of the endogenous KP DNA locus using PCR, followed by Sanger sequencing.

We constructed dual-labeled diploid strains that are heterozygous for the mTq2FP-tagged KP gene but homozygous for the YFP-tagged KP gene, so as to maximize the number of the acceptor YFPs and thus improve the chance for FRET to occur. To construct these strains, we first mated one haploid *MATα* strain containing the mTq2FP-tagged protein with another haploid *MAT* **a** strain containing the YFP-tagged protein. We selected the resulting diploid strains using the marker genes linked to the fluorophores, and we subsequently sporulated and dissected these to identify haploid spores. We then selected the *MATα* haploid spore that contained both the two FP-tagged KP genes and mated this with the earlier *MAT* **a** YFP-containing haploid strain and selected the desired diploid strain (**Suppl. Fig. S1b**) using auxotrophic markers (*trp1-1* and *lys2∆*). To create the donor-only strains and the strains where mTq2FP and YFP were stochastically tagged to the same KP, only the first mating and selection step was needed. We note that when making the strain with Ndc80p-mTq2FP and Nuf2p-YFP, and the strain with Nuf2p-mTq2FP and Ask1p-YFP, we only performed the first mating and selection step, as the two resulting diploid strains failed to sporulate.

As positive controls, we constructed four plasmid vectors expressing tandemly labeled KPs (KP-mTq2FP-YFP) and then transformed them into unlabeled diploid cells using the LiOAc method mentioned above.^66^ In each case, we used homologous recombination to repair a plasmid cut with a restriction enzyme with linear DNA encoding the KP and the tandem fluorophores; we confirmed the sequence of all constructs by Sanger sequencing.

### Sample Preparation for FLIM Experiments

For each FLIM experiment, we prepared up to 10 yeast cultures, one for each strain. We grew yeast cultures overnight at 23 °C in 5 mL of synthetic complete media (1.7 mg/mL yeast nitrogen base, 5 mg/mL ammonium sulfate, 109 µM adenine sulfate, 95 µM l-arginine sulfate, 95 µM l-histidine HCl, 229 µM l-isoleucine, 457 µM l-leucine, 164 µM l-lysine HCl, 134 µM l-methionine, 303 µM l-phenylalanine, 98 µM l-tryptophan, 166 µM l-tyrosine, 178 µM uracil, 1280 µM l-valine, and 2% glucose as the carbon source) plus 100 mg/mL of additional adenine (SC+ADE). We diluted the resulting stationary phase cultures 20× into fresh media and incubated them for a further ~5 h to reach log phase. We then transferred each culture into three separate wells of a 96-well plate ~1 h before imaging. We next placed these plates into the microscope chamber at room temperature (~24 °C) to allow the cells to settle for imaging.

### Automated Multiwell Plate FLIM Microscope and FLIM Data Acquisition

**Figure 2.**
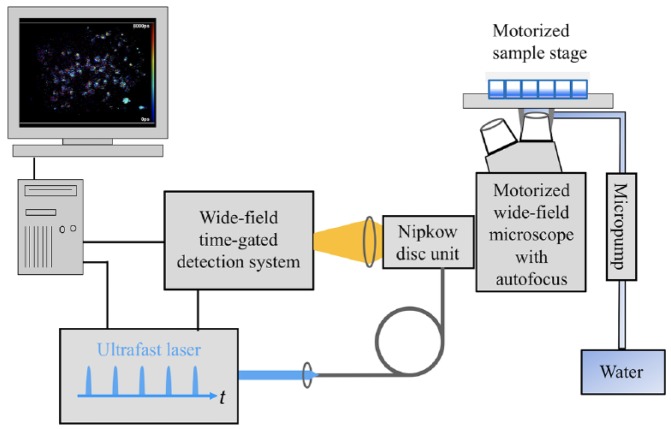
Schematic of the automated multiwell plate FLIM microscope system for FLIM HCA. This includes the electronically controlled micropump for maintaining the immersion water for the microscope objective during automated unsupervised imaging. Refer to the main text and Görlitz et al.^30^ for a detailed description of the system.

We constructed the instrument represented in **Figure 2** around a motorized inverted epifluorescence microscope frame (IX-81, Olympus, Tokyo, Japan) with ZDC autofocus, for which Görlitz et al.^30^ provide a detailed description of its operation. We arrayed the yeast cells in a glass-bottomed 96-well plate (SensoPlate, Grenier Bio-One GmbH, Germany), which we mounted on a motorized x-y stage (Märzhäuser Wetzlar GmbH, Germany). To provide optically sectioned FLIM, the instrument incorporated a spinning Nipkow disc unit (CSU-X1, Yokogawa Electric Corporation, Tokyo, Japan), as previously described.^30,67^ We used a 60× water immersion objective lens (UPlanSApo 60x, Olympus) with an NA of 1.2 for this study, noting that the close index matching resulted in improved imaging compared with using an oil immersion objective lens. We maintained the water immersion during the automated unsupervised imaging across the multiwell plate utilizing a micropump (mp6, Bartels Mikrotechnik GmbH, Dortmund, Germany) to continuously supply water to a retaining cap around the objective lens. This water immersion cap was provided by the European Molecular Biology Laboratory, Heidelberg, Germany. To provide the pulsed excitation radiation (80 MHz repetition rate, 434 nm central wavelength), we directed the output beam from a tunable femtosecond Ti:Sapphire laser (Mai Tai HP, Spectra-Physics, Harwell, UK) to a second harmonic generation unit (cat. 3980 Frequency Doubler/Pulse Selection Unit, Spectra-Physics, UK). The excitation pulses were passed through a 5 cm pathlength in a Brewster-angle cut quartz rod to increase the pulse width (and thereby reduce the peak intensity and nonlinear photobleaching) before being directed via a polarization-preserving single-mode optical fiber to the spinning Nipkow disc unit. We set the excitation power to be ~200 µW at the objective back focal plane. The fluorescence emission was imaged onto a gated optical intensifier (GOI; HRI-HL, Kentech Instruments Ltd., Wallingford, UK) via an emission filter (482/35 nm), and the resulting time-gated images at the phosphorus screen were imaged onto a cooled CCD camera (Orca II ER, Hamamatsu, Hamamatsu City, Japan) with a relay of 0.7× demagnification. We set the GOI gating voltage signal to 4 ns width and synchronized and delayed with respect to the laser excitation pulses. The delay between the excitation pulses and the gate was controlled using the µManager plugin openFLIM-HCA^30^ (see below). We chose 4 × 4 hardware binning at the CCD camera to further improve the S/N, and consequently each acquired image had 336 × 256 pixels and a pixel size of 615 nm. We typically acquired time-gated images of mTq2FP fluorescence at seven different delays with respect to the excitation pulses for FLIM (following the acquisition of five time-gated images prior to the FLIM data acquisition that pre-photobleached the background autofluorescence in the samples, as discussed in Supplemental Materials). We set the integration time of the CCD camera to 3 s per gate delay for mTq2FP donor FLIM acquisition, such that the full dynamic range of the CCD camera was utilized.

We utilized our previously reported µManager plugin for openFLIM-HCA^30^ to control the automated FLIM HCA data acquisition, imaging 15 FOVs within each well and acquiring FLIM data from all wells across the array without manual intervention. On average, it required ~45 s to image each FOV, and so an experiment investigating 10 strains, with 3 replicate wells per strain and 15 FOVs per well, required ~6 h for the whole FLIM HCA acquisition. We saved the FLIM HCA data as a series of OME-TIFF files (i.e., one TIFF file per FOV) in this study. More information and a detailed description of the µManager software can be found on the openFLIM-HCA wiki.^68^

For each experiment, we checked the proper functioning of the instrumentation by measuring the fluorescence decay of a reference dye (75 µM Coumarin 6 in ethanol) with temporal sampling at 25 ps intervals across the whole 12.5 ns pulse period. In addition, we measured the fluorescence decay profile of a 250 µM fluorescein solution in water quenched by 2 M potassium iodide, which exhibited a fluorescence lifetime of ~100 ps. We utilized this measurement and the measured Coumarin 6 decay profile to determine the instrument response function (IRF) via reference reconvolution^69–71^ for the subsequent FLIM data analysis.

### FLIM Data Processing and Lifetime Analysis

**Figure 3.**
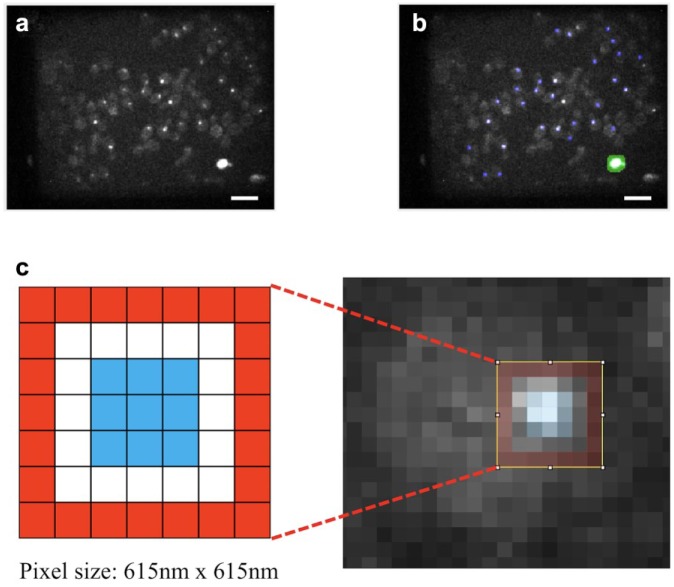
Strategy for segmenting FLIM images. (**a**) Exemplar image showing the fluorescence intensity integrated over the 12 time gates of a FLIM image of donor-only cells expressing Ask1p-mTq2FP. Note the appearance of the spot-like kinetochore clusters, the cellular autofluorescence (indicating the profiles of the cells), and the large bright dead cell. Scale bar: 20 µm. (**b**) Exemplar image showing the kinetochore segmentation mask (blue) and the dead cell exclusion mask (green) overlaid on the original image. We segmented the kinetochore clusters using a modified version of the NTH algorithm (see main text for details), whereas the dead cell was picked out by both its brightness and size. Scale bar: 20 µm. (**c**) Enlarged view of a segmented kinetochore region. Each kinetochore was segmented as a 3 × 3 pixel region (blue) centered on a pixel selected by the modified NTH algorithm. To remove the local cellular background for each kinetochore region, the median value from the pixels in a 7 × 7 hollow square (red) was subtracted off the pixel values in the kinetochore region in every temporal frame.

For each multiwell plate FLIM HCA dataset, we first processed the KP FLIM data using a MATLAB program custom written for budding yeast kinetochore data.^72^ This program sums the 12 time-gated images of each FLIM FOV dataset and subtracts the fixed background originating from the camera offset and ambient light to produce a fluorescence intensity image (**Fig. 3a**). We then automatically reject regions of dead cells in these intensity images based on their typical larger sizes and higher pixel intensities compared with the kinetochore foci (**Fig. 3b**). We then segment kinetochore foci using a modified version of the nonlinear top-hat (NTH) algorithm,^73^ which picks out regions brighter than the local background. This generates a segmentation mask covering the brightest pixel in each region and then dilates each segmented pixel into a 3 × 3 square. Therefore, each of the segmented kinetochore regions covered a square of approximately 3 × 3 pixels (**Fig. 3c**). For each time-gated fluorescence intensity image, the program then designates a 7 × 7 pixel hollow square with 1-pixel thickness as the local cellular background region for the kinetochore region at its center (red pixels in inset to **Fig. 3c**) and sets the local time-varying background (TVB) for each kinetochore focus to be the median pixel value from this cellular background region. This procedure results in a set of kinetochore-segmented, TVB-subtracted, time-gated fluorescence images, ready for subsequent lifetime analysis using the custom-written open-source software, FLIMfit,^31^ available at www.openmicroscopy.org/site/products/partner/flimfit. When fitting the measured fluorescence decay profiles to an exponential decay model, FLIMfit takes account of the contributions from earlier incomplete fluorescence decays and utilizes the IRF in the fitting. We measured the IRF for each experiment and determined its temporal drift across an FOV using “reference reconvolution” in FLIMfit, based on the finely sampled decays from a Coumarin 6 sample and a quenched fluorescein sample as mentioned above.

For the work reported here, we utilized the capability of FLIMfit to perform “image-wise” fitting, that is, to globally fit the decay data from all the kinetochore regions over a whole FOV to one decay model, which provides a higher fitting precision due to the increased photon number, compared with individually fitting each pixel (pixel-wise fitting) or each kinetochore region. We chose to fit the fluorescence lifetime data to a monoexponential decay model (see **Suppl. Fig. S3** for exemplar decay profiles and fitting results), since the relatively low number of time gates was insufficient to reliably fit to a more complex decay model, and so we are limited to detecting KP interactions rather than quantifying them. Having obtained a single lifetime value for each of the 45 FOVs imaged for each strain in each experiment, we then combined the fluorescence lifetime measurements of the same strains from separate experiments on different days to provide larger sample sizes in order to average over experimental noise and biological heterogeneity. We found that the precision and accuracy of the image-wise fluorescence lifetime measurements correlated with the signal level (i.e., number of detected photons per image). Consequently, we set a global threshold for the total signal per image (i.e., the number of kinetochore pixels multiplied by the mean integrated intensity value from those pixels for each image) to be greater than 1.2 × 10^5^ camera digital numbers, and we rejected data from images with total signal below this threshold.

We performed the above FLIM data processing and lifetime analysis procedures on a personal laptop (Intel Core CPU i7 at 2.670 GHz with four physical cores and 16 GB RAM). Fitting the image-wise lifetimes for a typical KP FLIM dataset for one yeast strain comprising images of 45 FOVs required <10 s.

### Statistics

We performed statistical comparisons between the lifetime results combined from all measurements of the query strain and the corresponding negative controls using the two-sided Wilcoxon rank-sum test. A significance level of α = 0.05 was used. In groups where there was more than one query strain, we applied a Bonferroni correction to α to correct for multiple comparisons.

## Results

### Strains and Plasmids Constructed for the Study

We constructed six groups of dual-labeled diploid strains and grouped the strains according to which of the six selected KPs (Ask1p, Ndc80p, Nuf2p, Spc24p, Mtw1p, Ndc10p) we had tagged by the donor mTq2FP. The six selected KPs are representative of KPs along the kinetochore–microtubule axis (**Fig. 1c**). In each group, there was at least one “query strain,” where we had labeled a different KP with the YFP acceptor for the FRET measurement, and one donor-only strain that served as a negative control for FRET. A reduction in the query strain donor fluorescence lifetime compared with the non-FRETing donor lifetime of the corresponding negative control was considered to indicate the presence of FRET. We note that mTq2FP presented slightly different non-FRETing donor lifetimes in the donor-only strains as it was linked to different KPs. This may be attributed to the different local molecular environments. Therefore, we only compared donor lifetimes between two strains in the same group, where the donor mTq2FP was tagged to the same KP. A list of these strains can be found in **Supplemental Table S1**.

Besides the endogenously labeled query and negative control strains, for four of the six selected KPs (Ask1p, Spc24p, Mtw1p, Ndc10p), we engineered a positive control plasmid that expressed a construct where one chosen KP was sequentially tagged by the FRET pair mTq2FP and YFP, as outlined in Materials and Methods. In each of these constructs, we tagged the KP with mTq2FP via a 4-alanine linker and we linked the donor mTq2FP to the acceptor YFP via an 8-amino-acid linker (four repeats of glycine-serine). This short flexible linker should ensure that mTq2FP and YFP undergo FRET. We transferred these plasmids into unlabeled diploid cells to serve as positive controls for FRET, thus allowing us to test whether we can detect any FRET between yeast KP using the optimized openFLIM-HCA platform. **Supplemental Table S2** lists these positive control plasmids.

We performed FLIM HCA experiments on all the constructed yeast strains. In each experiment, we imaged at least one group of strains (comprising the query, negative control, and positive control strains) using the automated multiwell plate FLIM microscope shown in **Figure 2**, and we analyzed the resulting datasets as described in Materials and Methods. To explore the reproducibility of these measurements, we imaged each group of strains in two to four separate multiwell plate experiments. For each strain, we combined the average (image-wise) lifetime values from different experiments (including only the images that passed the threshold for sufficient total signal) as shown in the box plots in the following subsections. The median, interquartile range, and 95% confidence interval of the measured image-wise lifetimes for each strain are listed in **Supplemental Table S3**.

### All Positive Controls Exhibited Significantly Reduced Donor Fluorescence Lifetimes Compared with the Corresponding Negative Controls

**Figure 4.**
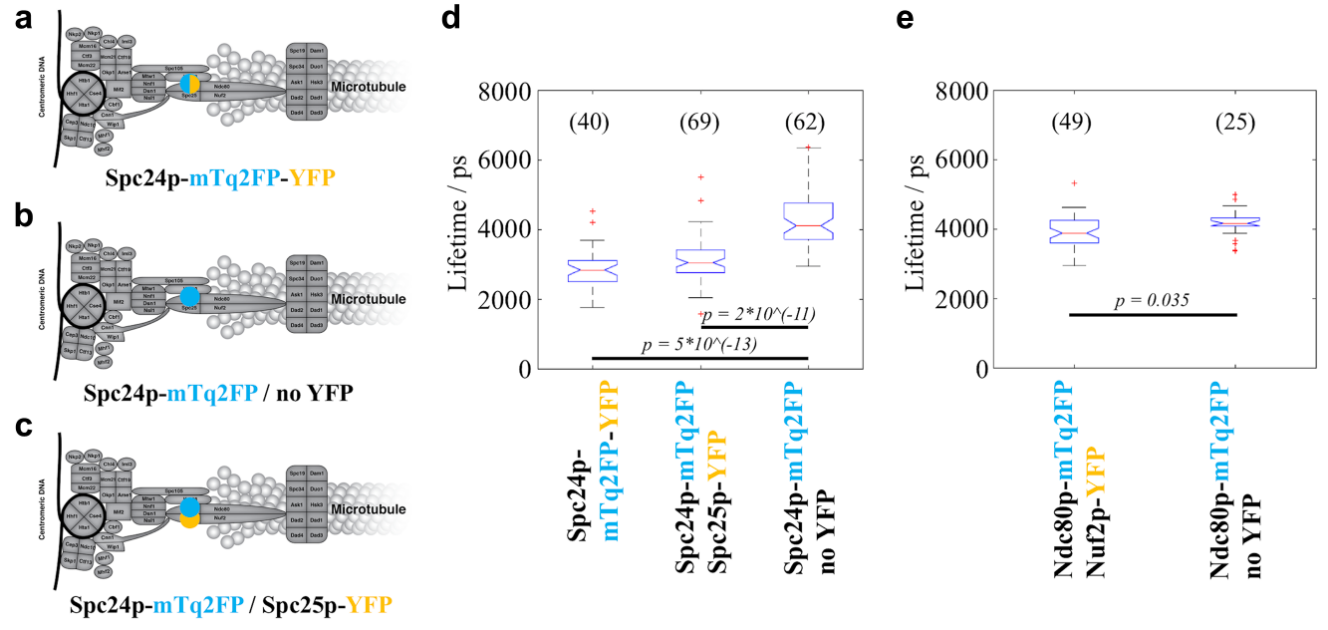
FLIM results of the strains in the Spc24p-mTq2FP and Ndc80p-mTq2FP groups. (**a–c**) Schematics of the positions of the fluorophores in the three strains in the Spc24p-mTq2FP group: (**a**) the positive control containing a plasmid expressing Spc24p-mTq2FP-YFP; (**b**) the donor-only negative control; and (**c**) the query strain where YFP was tagged to Spc25p, which was known to form a dimer with Spc24p in the NDC80 complex. (**d**) The query strain expressing Spc24p-mTq2FP and Spc25p-YFP exhibited a significantly reduced donor fluorescence lifetime compared with the negative control. (**e**) The query strain expressing Ndc80p-mTq2FP and Nuf2p-YFP exhibited a significantly reduced donor fluorescence lifetime compared with the negative control (note that this query strain is heterozygous for both FP-tagged KP genes). Each box plot shows the median value (bar) and the quartiles (box) of the image-wise lifetime values. The notches indicate the 95% confidence interval of the median. The whiskers correspond to the minimum of the data range or 1.5× the interquartile range, whereas extreme data points are marked as outliers. The number of FOVs analyzed for each strain is shown in parentheses above the corresponding box. The statistical test performed was the two-sided Wilcoxon rank-sum test. A Bonferroni correction was performed to correct the α value for multiple comparisons. *p* values smaller than the corrected α values are shown in the box plots.

**Figure 5.**
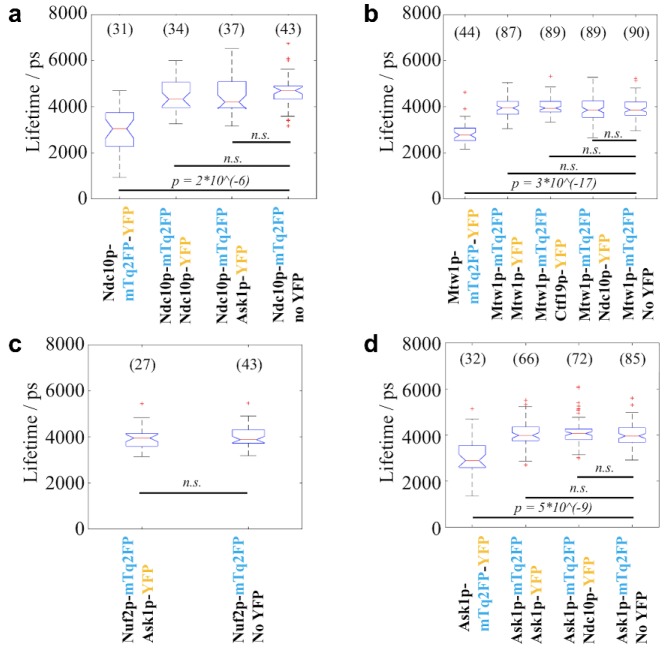
FLIM results of the strains in the (**a**) Ndc10p-mTq2FP, (**b**) Mtw1p-mTq2FP, (**c**) Nuf2p-mTq2FP, and (**d**) Ask1p-mTq2FP groups. None of the query strains in these groups exhibited a significantly changed lifetime compared with the donor-only negative controls. The statistical test performed was the two-sided Wilcoxon rank-sum test. A Bonferroni correction was performed to correct the α value for multiple comparisons. *p* values smaller than the corrected α values are shown in the box plots. n.s. = not significant.

For each of the four positive controls, we compared the lifetime of the donor fluorophore mTq2FP with that from a negative control strain that only expresses the donor tag linked to a KP. We found that, in each case, the lifetime of the donor fluorophore was significantly decreased when compared with the negative control (**Figs. 4 and 5**). These data show that our system is capable of detecting FRET between closely located donor and acceptor fluorophores at budding yeast kinetochores by measuring changes in the donor fluorescence lifetime.

### Two Query Strains Showed Significantly Reduced Donor Fluorescence Lifetimes Compared with the Negative Controls

Among the 10 query strains imaged, two showed donor fluorescence lifetimes significantly shorter than the corresponding negative controls, as tested by the Wilcoxon rank-sum test with Bonferroni correction. The first strain, encoding Spc24-mTq2FP and Spc25-YFP, exhibited a reduction in median donor lifetime of 1064 ps (**Fig. 4d**), while the median lifetime reduction for the second strain that expressed Ndc80p-mTurquoise2 and Nuf2p-YFP was 284 ps (**Fig. 4e**). Previous studies have revealed that Spc24p dimerizes with Spc25p while Ndc80p dimerizes with Nuf2p.^74–76^ Thus, our FLIM HCA results are consistent with the existence of these two dimers, indicating that our methodology is capable of detecting close proximity of endogenously tagged KPs. This could be used as a readout of the structural integrity of this compact kinetochore structure in live budding yeast cells.

### All Other Query Strains Showed No Difference in Donor Fluorescence Lifetime between the Corresponding Negative Controls

The measured lifetimes of the remaining eight query strains are shown in **Figure 5**. Compared with the results from the corresponding negative controls, the lifetimes did not show statistically significant differences. Among these query strains, FRET was expected for one strain where Ndc10p was stochastically labeled with mTq2FP and YFP, as it was previously reported that two copies of Ndc10p should dimerize near each centromere.^77,78^ The absence of a lifetime reduction in this strain (**Fig. 5a**) may suggest that the C-termini of the two copies of Ndc10p in each homodimer are not close enough for FRET to occur. Alternatively, it could be a false-negative result that could be due to both the stochastic labeling and the low copy number of this inner KP, as discussed below. For the other strains, however, FRET was not expected based on previous knowledge of the kinetochore structure. These results therefore suggest that our FLIM HCA methodology is largely free of false-positive results (**Fig. 5b–d**).

## Discussion

We have demonstrated a methodology for FLIM HCA to study interactions of low-copy-number proteins in live cells and have applied this to budding yeast kinetochores. For this we adapted our previously reported FLIM HCA platform^30^ for automated imaging on endogenous budding yeast KPs labeled by FPs, addressing the challenges of low S/N due to low endogenous copy numbers of KPs and strong cellular and background autofluorescence. For efficient excitation and collection of the fluorescence signal in live cells, we utilized a water immersion objective lens of NA 1.2, and we set the average excitation power to a level that balanced the requirements of detected signal level, imaging speed, and the impact of photobleaching. To minimize the (autofluorescence) background, which may be more severe when performing wide-field imaging compared with confocal or multiphoton microscopy, we utilized a spinning Nipkow disc unit to provide optical sectioning. We maximized the S/N by appropriately setting the gate width of the GOI and the spatial binning of the CCD. The custom openFLIM-HCA open-source µManager plugin^30^ and the automated feed of immersion water to the objective lens enabled unsupervised imaging of multiple strains in a single FLIM HCA experiment. Global fitting of the resulting datasets allowed us to obtain average FLIM measurements based on many FOVs, thus mitigating the effects of instrument noise and biological heterogeneity. A custom MATLAB-based algorithm enabled us to reliably exclude regions with dead cells, segment kinetochore clusters, and subtract the local TVB for each kinetochore cluster and generate TVB-subtracted images for lifetime analysis in the custom software FLIMfit.^31^ While we have focused on global fitting to undertake FLIM with relatively low photon numbers, we note that there are other successful approaches, including phasor analysis^79^ and Bayesian estimation of fluorescence lifetime parameters.^80^

To explore the capability of FLIM HCA to assay FRET between endogenous proteins labeled with FPs, we labeled six groups of diploid query strains with the mTq2FP/YFP FRET pair. In these query strains, we tagged the fluorophores to various KPs that are known to be either close to each other or distant. Since the fluorescence lifetime of a donor fluorophore is dependent on its microenvironment and the intrinsic lifetime of green fluorescent protein (GFP) in budding yeast cells varies when tagged to different proteins,^56^ we grouped the query strains according to which KP was tagged with mTq2FP and constructed negative control strains expressing only the mTq2FP donor-tagged KP (i.e., without any YFP acceptor). We also included positive FRET controls in some groups, which were diploid strains expressing constructs where a KP was tandemly tagged by both mTq2FP and YFP. We expressed the FP-tagged KPs from these positive control strains from plasmids driven by a *CUP1* promoter, since we suspected that doubly tagged KPs might cause problems for the function of the kinetochore if present endogenously. Plasmids that ectopically express FP-tagged KPs under a *CUP1* promoter contribute typically half of the total number of that specific KP at the kinetochore in a diploid strain.^81^

In line with our expectations, the FLIM results presented reduced donor lifetimes for all positive control yeast strains relative to the negative controls and also in two query strains with fluorescently tagged KPs in the well-characterized Ndc80 complex. This complex at the outer kinetochore comprises four components, Ndc80p, Nuf2p, Spc24p, and Spc25p, which form two dimers (Ndc80p-Nuf2p and Spc24p-Spc25p) where the C-termini of these proteins in each dimer are in close proximity.^52,74,75^ Our FLIM results are consistent with these data. We note that previous ratiometric FRET measurements also indicated that the C-termini of Ndc80p and Nuf2p are close to each other and those of Spc24p and Spc25p may be closer.^49,50^ However, Aravamudhan et al.^52^ suggest that the FRET efficiency for Ndc80/Nuf2p should be higher than we observe. This discrepancy may be partly explained by our use of a heterozygously tagged strain, where ~50% of the Nuf2p proteins are not tagged by YFP, thus resulting in a lower measured “effective FRET efficiency.”

The other query strains where the donor and the acceptor were tagged to different pairs of KPs presented no significant change in lifetime compared with the negative controls. We expected these results, as each of the pairs of KPs that the mTq2FP/YFPs tagged are well separated along the kinetochore–microtubule axis (e.g., Ask1p-mTq2FP/Ndc10p-YFP) (see the review by Cieśliński and Ries and references therein^50^), and this indicates that neither intrakinetochore FRET nor interkinetochore FRET occurred between the fluorophores. Moreover, these data also suggest that our methodology can consistently reveal the non-FRETing lifetimes, even for KPs that have copy numbers as low as ~2–4 per kinetochore.

We did not observe a significant change in lifetime for the two strains in which Mtw1p and Ndc10p were stochastically tagged with both mTq2FP and YFP (e.g., one allele encoding Mtw1p-mTq2FP and the other Mtw1p-YFP). For the strain with stochastically tagged Mtw1p, the non-FRETing result is supported by a previous intensity-based FRET study that showed that while the N-termini of Mtw1p molecules in one kinetochore may be in close proximity, the C-termini were further apart, likely precluding FRET.^52^ However, we did expect the query strain that expressed stochastically tagged Ndc10p to present a donor lifetime change indicating FRET, as the inner KP Ndc10p is expected to form a dimer proximal to the centromere. The absence of FRET may be explained by the C-termini of the Ndc10p homodimer being separated by >10 nm, which may be supported by the structure of Ndc10p reported for another yeast species, *Kluyveromyces lactis*.^80^ However, it has also been suggested that the dimerization domain of budding yeast Ndc10p is in its C-terminus, as dimerization was not seen for the N-terminal halves of the protein.^82^ An alternative explanation is that this is a false-negative result due to low S/N, arising because there is currently thought to be only one Ndc10p dimer at each centromere.^78^ Since each Ndc10p dimer was stochastically labeled with FRET fluorophores, on average only 50% of the imaged donors would undergo FRET—and there could be further Ndc10p molecules in the kinetochore clusters but located outside the inner kinetochore CBF3 complex,^83^ which may not form dimers and therefore would reduce the measured FRET signal.

For the mTq2FP/YFPs used here, the interquartile ranges of donor lifetimes of all the strains were 240–1450 ps. With the numbers of FOV taken for the various strains as shown in **Figures 4 and 5**, the resulting 95% confidence intervals of the median lifetimes were in the range of 75–410 ps (calculated under the assumption of normal distribution^84^). While the statistical power for revealing differences in the donor fluorescence lifetime may be improved by increasing the sample size, we anticipate limited improvement since the uncertainty would decrease with the square root of the sample size and it may not be practical to image many more FOVs without compromising the fitness of the cells during such a prolonged experiment. Consequently, in order to optimize the power of this FLIM HCA methodology for identifying potentially interacting proteins, one should seek to maximize the potential donor lifetime change and/or to maximize the S/N through judicious choice of fluorophores. For example, these assays could be improved by employing brighter donor FPs that emit in a spectral window with less overlap with the cellular autofluorescence (such as enhanced GFP [EGFP]). Ideally, the FPs used for these FLIM FRET experiments should also have optimized maturation rates for yeast cultures at 23 °C, and they should be linked to the target KPs with flexible linkers to provide broader, more random distributions of fluorophore orientations. In summary, we have demonstrated that the automated FLIM HCA methodology described here offers a means to systematically study interactions of endogenous proteins in compact budding yeast kinetochores. We aim to apply this method to study the dynamic kinetochore structure in the presence/absence of microtubule-generated tensions, as well as to investigate the interactions between KPs and regulatory proteins for mitotic progression, for a better understanding of the interplay among kinetochores, microtubule attachment, and faithful chromosome segregation.

## Supplemental Material

Supplemental_Material_819240 – Supplemental material for Automated Fluorescence Lifetime Imaging High-Content Analysis of Förster Resonance Energy Transfer between Endogenously Labeled Kinetochore Proteins in Live Budding Yeast Cells Click here for additional data file. Supplemental material, Supplemental_Material_819240 for Automated Fluorescence Lifetime Imaging High-Content Analysis of Förster Resonance Energy Transfer between Endogenously Labeled Kinetochore Proteins in Live Budding Yeast Cells by Wenjun Guo, Sunil Kumar, Frederik Görlitz, Edwin Garcia, Yuriy Alexandrov, Ian Munro, Douglas J. Kelly, Sean Warren, Peter Thorpe, Christopher Dunsby and Paul French in SLAS Technology
