# Supplementary material for: Automated Fluorescence Lifetime Imaging High-Content Analysis of Förster Resonance Energy Transfer between Endogenously Labeled Kinetochore Proteins in Live Budding Yeast Cells
Source: SLAS Technol. 2019 Jan 10;24(3):308–20. doi: 10.1177/2472630318819240 (PMC6537140; doi:10.1177/2472630318819240)
Supplement: Supplemental_Material_819240 – Supplemental material for Automated Fluorescence Lifetime Imaging High-Content Analysis of Förster Resonance Energy Transfer between Endogenously Labeled Kinetochore Proteins in Live Budding Yeast Cells [file Supplemental_Material_819240.pdf]

Supplementary Material

Automated fluorescence lifetime imaging high content analysis of Förster resonance energy transfer between endogenously-labeled kinetochore proteins in live budding yeast cells

Wenjun Guo[1,2], Sunil Kumar[1,2], Frederik Görlitz[1], Edwin Garcia[1], Yuriy Alexandrov[1,2], Ian Munro, Douglas J. Kelly[1,3], Sean Warren[4], Peter Thorpe[2,5], Christopher Dunsby[1,2,6]*, Paul French[1,2]*

* Denotes equal contribution to this work

[1] Photonics Group, Department of Physics, Imperial College London, London SW7 2AZ, UK
[2] Francis Crick Institute, 1 Midland Road London NW1 1AT, UK
[3] RIKEN Center for Biodynamic Systems Research, Kobe, Japan
[4] Garvan Institute of Medical Research, University of New South Wales, Sydney, Australia
[5] Queen Mary University of London, Mile End Road, London E1 4NS, UK
[6] Centre for Pathology, Imperial College London, Du Cane Rd, London W12 0NN, UK

Corresponding author: Paul French (paul.french@imperial.ac.uk)

Table S1: Yeast strains used for the FLIM experiments

Strain	Genotype (genotypes listed once are homozygous)	Source
PT208	*MATα/MAT***a** *ADE2 RAD5 TRP1/trp1-1 LYS2/lys2Δ his3-11,15 leu2-3,112 ura3-1 can1-100*	This work
PT267	*MATα/MAT***a** *ADE2 RAD5 TRP1/trp1-1 LYS2/lys2Δ his3-11,15 leu2-3,112 ura3-1 can1-100 SPC24-mTq2FP::HYGMX/SPC24 SPC25-YFP::HIS3MX6*	This work
PT244	*MATα/MAT***a** *ADE2 RAD5 TRP1/trp1-1 LYS2/lys2Δ his3-11,15 leu2-3,112 ura3-1 can1-100 SPC24-mTq2FP::HYGMX/SPC24*	This work
PT273	*MATα/MAT***a** *ADE2 RAD5 TRP1/trp1-1 LYS2/lys2Δ his3-11,15 leu2-3,112 ura3-1 can1-100 NDC80-mTq2FP::HYGMX/NDC80 NUF2-YFP::HIS3MX6/NUF2*	This work
PT278	*MATα/MAT***a** *ADE2 RAD5 TRP1/trp1-1 LYS2/lys2Δ his3-11,15 leu2-3,112 ura3-1 can1-100 NDC80-mTq2FP::HYGMX/NDC80*	This work
PT197	*MATα/MAT***a** *ADE2 RAD5 TRP1/trp1-1 LYS2/lys2Δ his3-11,15 leu2-3,112 ura3-1 can1-100 NDC10-mTq2FP::HYGMX/NDC10-YFP::HIS3MX6*	This work
PT270	*MATα/MAT***a** *ADE2 RAD5 TRP1/trp1-1 LYS2/lys2Δ his3-11,15 leu2-3,112 ura3-1 can1-100 NDC10-mTq2FP::HYGMX/NDC10 ASK1-YFP::HIS3MX6*	This work
PT261	*MATα/MAT***a** *ADE2 RAD5 TRP1/trp1-1 LYS2/lys2Δ his3-11,15 leu2-3,112 ura3-1 can1-100 NDC10-mTq2FP::HYGMX/NDC10*	This work
PT194	*MATα/MAT***a** *ADE2 RAD5 TRP1/trp1-1 LYS2/lys2Δ his3-11,15 leu2-3,112 ura3-1 can1-100 MTW1-mTq2FP::HYGMX/MTW1-YFP::HIS3MX6*	This work
PT268	*MATα/MAT***a** *ADE2 RAD5 TRP1/trp1-1 LYS2/lys2Δ his3-11,15 leu2-3,112 ura3-1 can1-100 MTW1-mTq2FP::HYGMX/MTW1 CTF19-YFP::HIS3MX6*	This work
PT269	*MATα/MAT***a** *ADE2 RAD5 TRP1/trp1-1 LYS2/lys2Δ his3-11,15 leu2-3,112 ura3-1 can1-100 MTW1-mTq2FP::HYGMX/MTW1 NDC10-YFP::HIS3MX6*	This work
PT260	*MATα/MAT***a** *ADE2 RAD5 TRP1/trp1-1 LYS2/lys2Δ his3-11,15 leu2-3,112 ura3-1 can1-100 MTW1-mTq2FP::HYGMX/MTW1*	This work
PT276	*MATα/MAT***a** *ADE2 RAD5 TRP1/trp1-1 LYS2/lys2Δ his3-11,15 leu2-3,112 ura3-1 can1-100 NUF2-mTq2FP::HYGMX/NUF2 ASK1-YFP::HIS3MX6/ASK1*	This work
PT279	*MATα/MAT***a** *ADE2 RAD5 TRP1/trp1-1 LYS2/lys2Δ his3-11,15 leu2-3,112 ura3-1 can1-100 NUF2-mTq2FP::HYGMX/NUF2*	This work
PT192	*MATα/MAT***a** *ADE2 RAD5 TRP1/trp1-1 LYS2/lys2Δ his3-11,15 leu2-3,112 ura3-1 can1-100 ASK1-mTq2FP::HYGMX/ASK1-YFP::HIS3MX6*	This work
PT266	*MATα/MAT***a** *ADE2 RAD5 TRP1/trp1-1 LYS2/lys2Δ his3-11,15 leu2-3,112 ura3-1 can1-100 ASK1-mTq2FP::HYGMX/ASK1 NDC10-YFP::HIS3MX6/NDC10*	This work
PT258	*MATα/MAT***a** *ADE2 RAD5 TRP1/trp1-1 LYS2/lys2Δ his3-11,15 leu2-3,112 ura3-1 can1-100 ASK1-mTq2FP::HYGMX/ASK1*	This work

Table S2: Yeast plasmids used for positive controls

Strain	Genotype	Source
pHT446	*pCUP1 ASK1-mTq2FP-YFP LEU2*	This work
pHT448	*pCUP1 MTW1-mTq2FP-YFP LEU2*	This work
pHT449	*pCUP1 NDC10-mTq2FP-YFP LEU2*	This work
pHT522	*pCUP1 SPC24-mTq2FP-YFP LEU2*	This work

Table S3: Summary of the image-wise fluorescence lifetime results for all yeast strains measured

Strain	No. of FOV	Median (ps)	Inter-quartile range (ps)	95% confidence interval (ps)
Spc24p-mTq2FP-YFP	40	2842	612	2842 ± 152
Spc24p-mTq2FP Spc25p-YFP	69	3052	654	3052 ± 124
Spc24p-mTq2FP No YFP	62	4116	1050	4116 ± 209
Ndc80p-mTq2FP Nuf2p-YFP	49	3886	645	3886 ± 145
Ndc80p-mTq2FP No YFP	25	4170	241	4170 ± 76
Ndc10p-mTq2FP-YFP	31	3037	1452	3037 ± 409
Ndc10p-mTq2FP Ndc10p-YFP	34	4323	1120	4323 ± 302
Ndc10p-mTq2FP Ask1p-YFP	37	4204	1133	4204 ± 292
Ndc10p-mTq2FP No YFP	43	4705	585	4705 ± 140
Mtw1p-mTq2FP-YFP	44	2762	539	2762 ± 128
Mtw1p-mTq2FP Mtw1p-YFP	87	3944	571	3944 ± 96
Mtw1p-mTq2FP Ctf19p-YFP	89	3939	458	3939 ± 76

Mtw1p-mTq2FP Ndc10p-YFP	89	3852	706	3852 ± 117
Mtw1p-mTq2FP No YFP	90	3863	582	3863 ± 96
Nuf2p-mTq2FP Ask1p-YFP	27	3948	545	3948 ± 165
Nuf2p-mTq2FP No YFP	43	3878	583	3878 ± 140
Ask1p-mTq2FP-YFP	32	2884	947	2884 ± 263
Ask1p-mTq2FP Ask1p-YFP	66	3983	607	3983 ± 117
Ask1p-mTq2FP Ndc10p-YFP	72	4065	457	4065 ± 85
Ask1p-mTq2FP No YFP	85	3955	637	3955 ± 108

Photobleaching characteristics of the fluorescence signals from kinetochore regions and cellular background

The data analysis entails subtracting a time-varying background (TVB) from the fluorescence signal for each kinetochore focus. For the excitation powers used, the cellular autofluorescence was found to photobleach much more significantly during an acquisition than the signal from the fluorescent proteins (FP). As illustrated in Supplementary figure S2, the photobleaching of the total signal (K) measured from the FP was approximately exponential, decreasing by ~40% over 120 s. After subtraction of the autofluorescence background (C), the net FP signal (K-C) was bleached by only ~7% during the 21 s measurement time of a typical FLIM acquisition, which was made after an initial 15 s period of pre-photobleaching. This was implemented by initially acquiring 5 time-gated images, so that the S/N of the time-gated images was subsequently more consistent for each FLIM measurement. These 5 initial time-gated images were excluded when performing fluorescence lifetime analysis but were included when calculating the integrated fluorescence intensity images used for image segmentation.

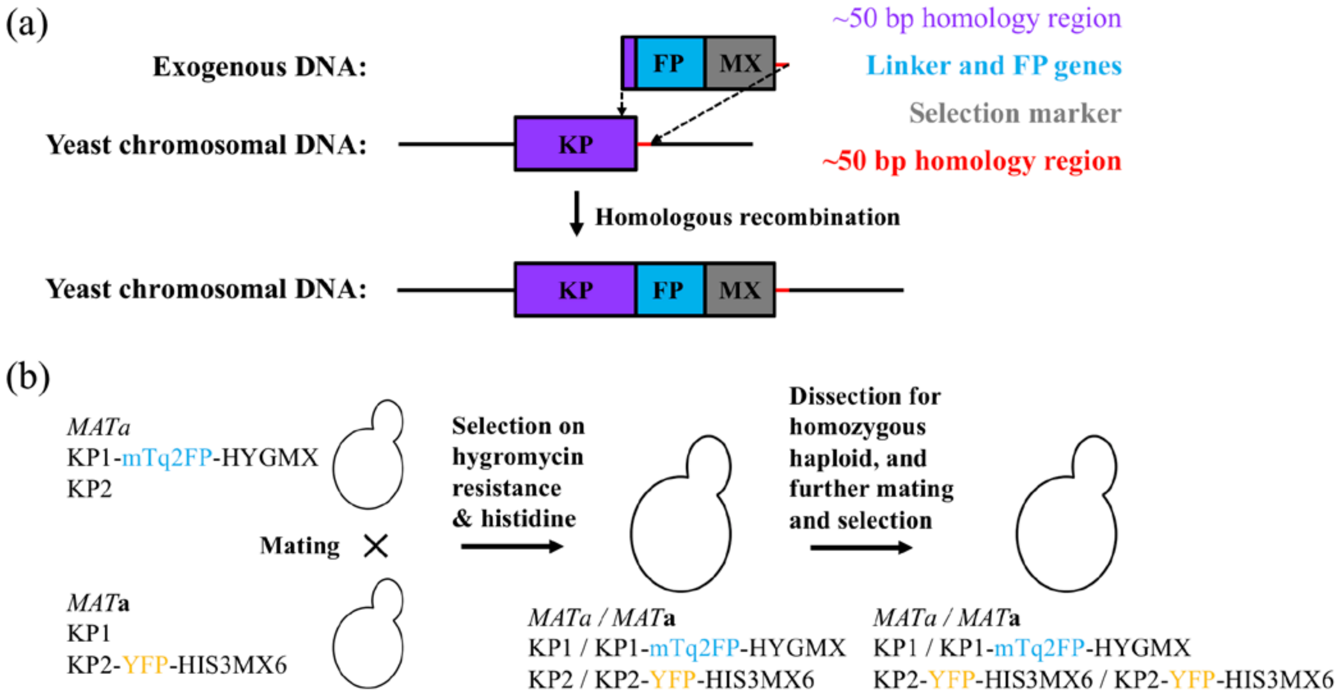

Supplementary figure S1: Strategies for constructing the endogenously-labelled yeast strains. (a) Endogenous labelling of each haploid yeast strain was achieved by performing yeast transformation using the LiOAc method to introduce the exogenous DNA containing the genes of the linker, the FP (mTq2FP or YFP), and the selection marker into yeast cells and then inserting it into the yeast genome via homologous recombination. (b) Each dual-labelled diploid strain that is heterozygous for the mTq2FP-tagged KP gene but homozygous for the YFP-tagged KP gene was constructed in a few steps. First, two endogenously-labelled haploid strains of opposite mating types were mated and selected for the resulting diploid strain that is heterozygous for both FP genes. Following that, the cells of that diploid strain were induced to sporulate into asci, each of which contained 4 haploid spores. The asci were then dissected, and a dual-labelled haploid spore with the desired mating type and auxotrophic markers was selected. Finally, the selected dual-labelled haploid was mated with the haploid containing the donor gene that was used in the first step, and a further selection step was performed for the desired diploid strain.

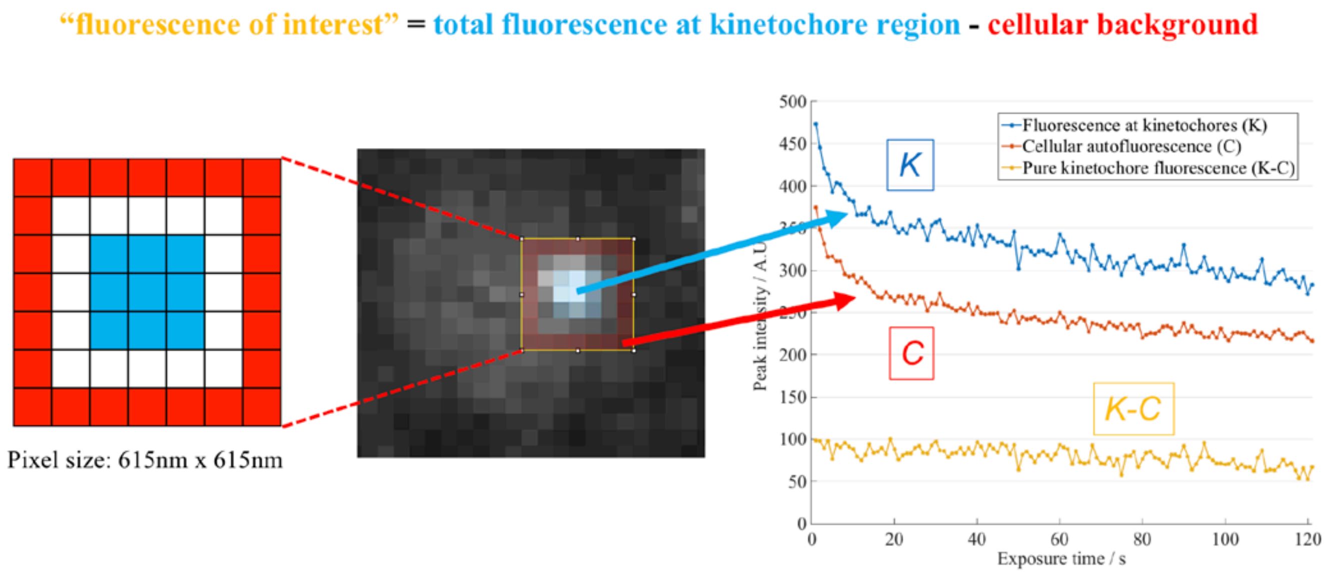

Supplementary figure S2. Photobleaching characteristics of various fluorescence signals from the FLIM data of yeast cells. The photobleaching curves (right) show the fluorescence signals from the kinetochore regions (blue), from the cellular background (red) and from the donor FPs at the kinetochore regions (yellow; equal to blue minus red), of the donor-only cells expressing Ask1p-mTq2FP. These FLIM data were acquired using an excitation power of 200 µW at the sample plane. The strategy for segmenting the kinetochore regions and the cellular background regions for this figure was the same as used for the FLIM analysis (see Materials and Methods). These photobleaching curves were obtained by sequentially acquiring 121 images with the time delay set for the peak fluorescence intensity, each with 1 s camera exposure time, and averaging signals from cells within 5 FOVs. These curves illustrate the photobleaching dynamics under continuous exposure to the excitation radiation for 2 minutes, showing that the rapid decrease in fluorescence (K) from the kinetochore regions at the beginning of the exposure seems to be due to the photobleaching of the cellular background (C) that is also present at the kinetochore regions. The fluorescence signal of interest from the donor FPs at the kinetochore regions (i.e. the difference between the KP fluorescence and the cellular background fluorescence) was relatively stable over the 120 s observation time. These photobleaching curves indicate that acquiring images for ~15 s prior to the FLIM data acquisition (green box) for each FOV of yeast cells should pre-photobleach the cellular background to provide a more stable S/N for the FLIM data acquisitions.

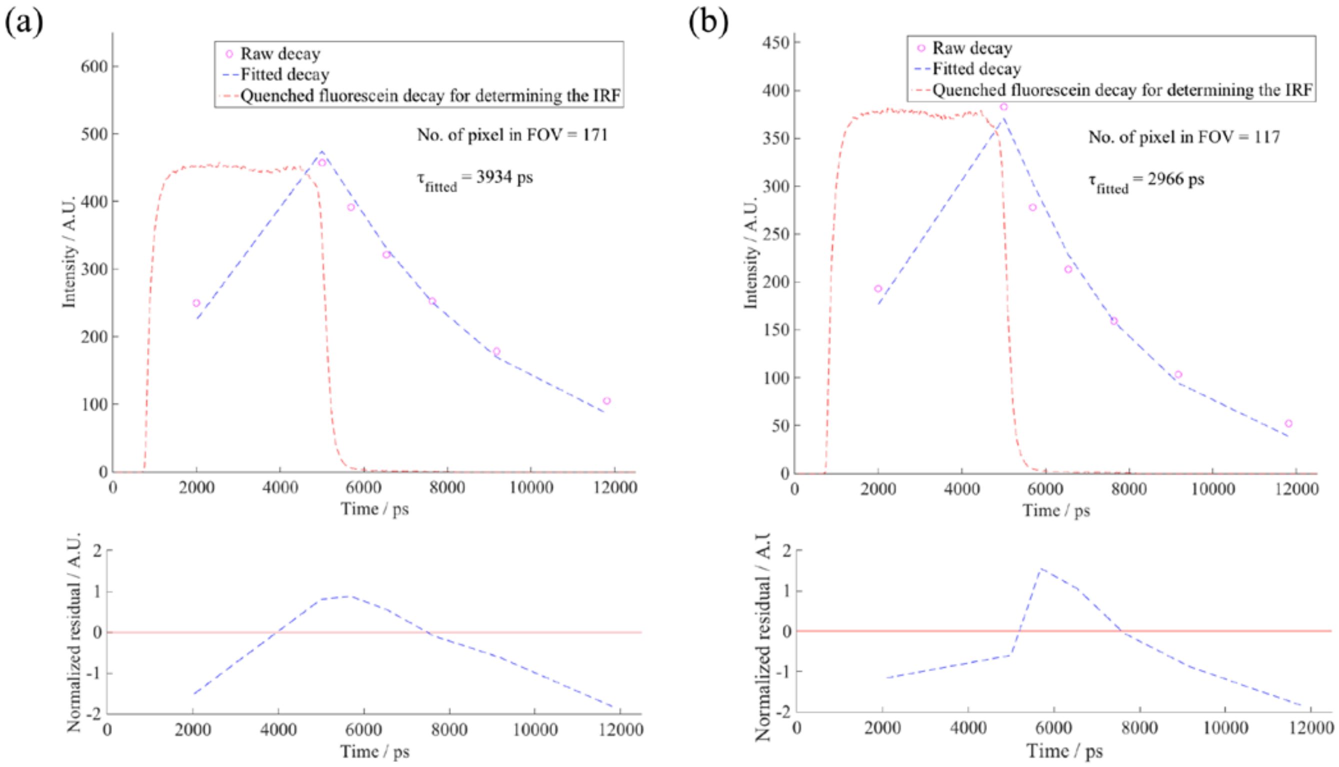

Supplementary figure S3. Exemplar image-wise raw donor fluorescence decay profiles and fitting results. (a) Image-wise mono-exponential fitting for the fluorescence decay measured from one typical FOV of cells expressing Spc24p-mTq2FP and no YFP. (b) Image-wise mono-exponential fitting for the fluorescence decay measured from one typical FOV of cells expressing Spc24p-mTq2FP and Spc25p-YFP.
